# Microbiome and PCOS: State-of-Art and Future Aspects

**DOI:** 10.3390/ijms22042048

**Published:** 2021-02-19

**Authors:** Pierluigi Giampaolino, Virginia Foreste, Claudia Di Filippo, Alessandra Gallo, Antonio Mercorio, Paolo Serafino, Francesco Paolo Improda, Paolo Verrazzo, Giuseppe Zara, Cira Buonfantino, Maria Borgo, Gaetano Riemma, Chiara De Angelis, Brunella Zizolfi, Giuseppe Bifulco, Luigi Della Corte

**Affiliations:** 1 Department of Public Health, University of Naples Federico II, 80131 Naples, Italy; pgiampaolino@gmail.com (P.G.); alessandra_gallo@hotmail.it (A.G.); francescopaolo.improda@gmail.com (F.P.I.); paoloverrazzo@gmail.com (P.V.); cirabuonfantino@gmail.com (C.B.); maria_borgo@hotmail.it (M.B.); m.chiaradeangelis@gmail.com (C.D.A.); brunella.zizolfi@hotmail.it (B.Z.); 2 Department of Neuroscience, Reproductive Sciences and Dentistry, School of Medicine, University of Naples Federico II, 80131 Naples, Italy; virgi.foreste@hotmail.it (V.F.); claudifilippo@gmail.com (C.D.F.); antoniomercorio@gmail.com (A.M.); paolo.serafino1992@gmail.com (P.S.); giuseppe.zara1404@gmail.com (G.Z.); giuseppe.bifulco@unina.it (G.B.); 3 Obstetrics and Gynecology Unit, Department of Woman, Child and General and Specialized Surgery, University of Campania “Luigi Vanvitelli”, 80131 Naples, Italy; gaetano.riemma7@gmail.com

**Keywords:** PCOS, microbiome, insulin-resistance, sexual hormones, therapeutic strategies

## Abstract

Polycystic ovary syndrome (PCOS) is a complex and heterogeneous endocrine disease. The hypothesis that alterations in the microbiome are involved in the genesis of PCOS has been postulated. Aim of this review is to summarize the available literature data about the relationship between microbiome and PCOS. A search on PubMed and Medline databases was performed from inception to November 20Most of evidence has focused on the connection of intestinal bacteria with sex hormones and insulin-resistance: while in the first case, a relationship with hyperandrogenism has been described, although it is still unclear, in the second one, chronic low-grade inflammation by activating the immune system, with increased production of proinflammatory cytokines which interfere with insulin receptor function, causing IR (Insulin Resistance)/hyperinsulinemia has been described, as well as the role of gastrointestinal hormones like Ghrelin and peptide YY (PYY), bile acids, interleukin-22 and Bacteroides vulgatus have been highlighted. The lower genital tract microbiome would be affected by changes in PCOS patients too. The therapeutic opportunities include probiotic, prebiotics and synbiotics, as well as fecal microbiota transplantation and the use of IL-22, to date only in animal models, as a possible future drug. Current evidence has shown the involvement of the gut microbiome in PCOS, seen how humanized mice receiving a fecal transplant from women with PCOS develop ovarian dysfunction, immune changes and insulin resistance and how it is capable of disrupting the secondary bile acid biosynthesis. A future therapeutic approach for PCOS may involve the human administration of IL-22 and bile acid glycodeoxycholic acid.

## 1. Introduction

Polycystic ovary syndrome (PCOS) is the most common cause of female endocrine infertility, with high heterogeneity and complexity, characterized by increased ovarian androgen biosynthesis, anovulation, and, as mentioned, infertility [1,2]; however, several aspects of women’s overall health are affected by PCOS, with long-term effects that transcend well beyond the reproductive age [3,4,5]. The prevalence of PCOS varies according to the population studied and the diagnostic criteria used, ranging from 8% to 13% [6]. Hyperandrogenism (HA) is one of the principal disorders that could characterize PCOS. Its excessive production by the ovaries or the adrenal cortex results in dermatological abnormalities (acne, hirsutism, androgenic alopecia), alteration of the hypothalamus-pituitary-gonadal axis and dysregulation of ovarian folliculogenesis that leads to menstrual disorder and infertility [6]. In 1992 the National Institutes of Health (NIH) defined PCOS by two features: (1) chronic anovulation, with (2) clinical and/or biochemical hyperandrogenism, in absence of other diseases (congenital adrenal hyperplasia, androgen-secreting neoplasms, thyroid dysfunction and hyperprolactinemia [7]. In 2003, the meeting of experts in Rotterdam revised the criterion; according to the European Society for Human Reproduction and Embryology/American Society for Reproductive Medicine (ESHRE/ASRM) PCOS is defined by the presence of 2 out of the following 3 features: oligo- and/or anovulation, clinical and/or biochemical signs of hyperandrogenism, and/or polycystic ovarian morphology (PCOM). The presence of 12 or more follicles measuring 2–9 mm throughout the entire ovary or an ovarian volume = 10 cm^3^ defines the PCOM [8,9].

The etiology of PCOS is still unclear; however, several factors have been identified, involved in generating a hormonal and metabolic imbalance that can lead to the development of this syndrome [9,10,11]. The most accredited theory is that of Frank et al. who considers PCOS a genetically determined disease whose clinical and biochemical heterogeneity depends on the interaction between genetic and environmental factors [12]. In the etiological hypotheses proposed over the years, the fact that, besides gynecological problems, PCOS can also lead to systemic metabolic disorders (such as hyperinsulinemia and insulin resistance (IR), obesity, increased risk of type II diabetes, cardiovascular disease) has played a fundamental role [13]. The numerous evidence on the correlation between the intestinal microbiome and the development of metabolic disorders, have led to postulate the hypothesis that alterations in the microbiome are also involved in the genesis of PCOS [14,15,16]. In 2012 the Dysbiosis of gut microbiota (DOGMA) hypothesis suggested that, following imbalance of intestinal flora, an increase of intestinal permeability could cause leakage of lipopolysaccharide (LPS) into the systemic circulation. The result is the activation of the immune system and inflammatory response that leads to IR [17]. Furthermore, recent studies are beginning to consider also the role of vaginal microbiome [18]. In consideration of the growing interest in the literature for the role of the microbiome in the genesis of numerous diseases, we try to summarize the main evidence published in literature regarding the relationship between microbiome and PCOS.

## 2. Materials and Methods

A literature search on the MEDLINE database (accessed through PubMed) for articles in English and published from inception to November 2020 was performed.

**Figure 1:**
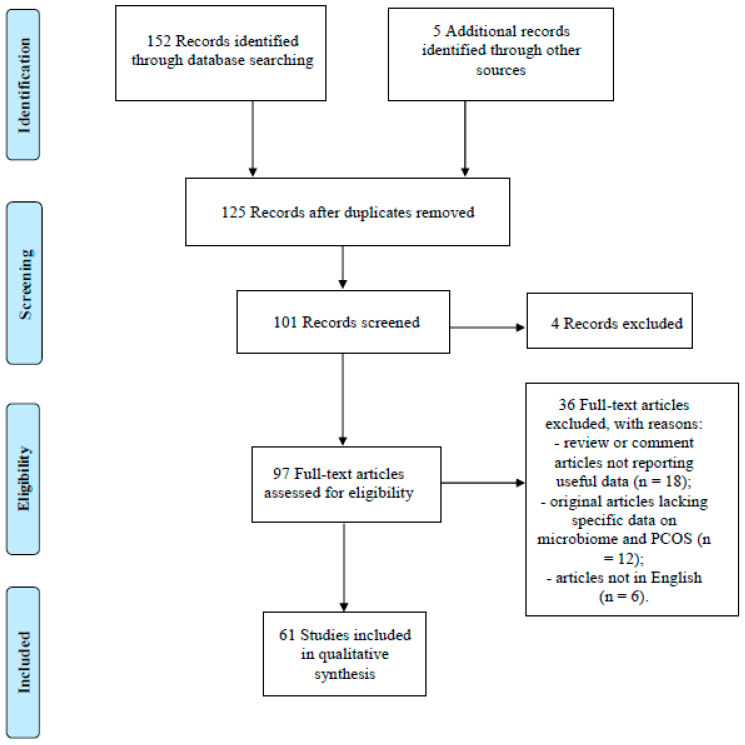
Flow diagram of narrative review search.

The following Medical Subject Headings (MeSH) terms were used to screen and identify studies: “PCOS”, “microbiome”, “molecular mechanism”, “therapy”, “insulin-resistance”, “sexual hormones” and “therapeutic strategies”. Non-English articles, published as conference papers or abstract only, and studies including information that overlapped other publications were excluded. In our search, only articles concerning PCOS were included. The selection criteria for the narrative review included original articles (randomized and non-randomized clinical trials, including prospective observational studies, retrospective cohort studies, and case-control studies) and review articles regarding the influence of PCOS on the microbiome. Articles that met the inclusion criteria were carefully read, and, when appropriate, further articles retrieved from their references were also reviewed with the aim to include other critical studies that might have been missed in the initial search. We presented here a narrative synthesis of the available evidence about the topic. Figure 1 reports the flow chart of article selection. 

## 3. Gut Microbiome and PCOS 

### 3.1. Gut Microbiome Changes in Women with PCOS

The human gut microbiome is made up of about 10^13^ to 10^14^ microorganisms, greater than 1000 different species and more than 7000 different strains [19,20]. The most represented microorganisms are bacteria, especially anaerobes, but the intestinal microbiome also including viruses, protozoa, archaea, and fungi [19]. The two most represented types of bacterial are *Bacteroidetes* and *Firmicutes* with *Proteobacteria*, *Actinobacteria*, *Fusobacteria*, and *Verrucomicrobia phyla* present in relatively low abundance. Gut microbiota plays a major role by influencing physiology, metabolism, nutrition, and immune function and, under physiological conditions, the delicate balance between the latter and the host prevents the development of different diseases [21,22]. There are significant differences in the composition of the microbiome between healthy adults, and these differences may underlie the susceptibility to various diseases [23]. The relationship between PCOS and gut microbiota changes has been the subject of numerous studies in recent years, that showed a significant difference in composition of gut microbiome between PCOS patients and healthy controls [24,25,26,27]. α and β diversity defines the microbiome changes: alpha (α) diversity is considered as an index of the health of an ecosystem and indicates the number of species present in a community that occupy a given environment in a specific community, whereas beta (β) diversity represents how similar one community is to another [28,29]. Several studies have reported a change in α and β diversity in patients with PCOS [25,26,30,31]. In addition to an alteration in the general composition of the microbiome, the same studies have shown that in PCOS there is also an alteration in balance of some species of bacteria, like *Bacteroidetes* and *Firmicutes* [24,25,26,27]; this modification can lead to altered production of short-chain fatty acids with a negative impact on metabolism, gut barrier integrity, and immunity [28]. Regarding the genera *Bacteroides*, Liu et al. have observed a specific increase of *Escherichia* and *Shigella* in women with PCOS, and a general gut microbiome composition similar respect that of obese control women [24]. Qi et al., instead, reported an increased abundance of Bacteroides vulgatus, accompanied by a reduction in the levels of glycodeoxycholic and tauroursodeoxycholic acid that leads to alteration of IL-22 level [31]. Moreover, Prevotella species abundance can be altered: its increase in PCOS patients arguing that may induce an adverse inflammatory effect to the host has been reported [32,33]. Another study, instead, founded a decrease of Prevotellaceae with a negative effect due to the loss of production of anti-inflammatory metabolites [34]. The beneficial bacteria, as *Lactobacilli* and *Bifidobacteria*, who enhancing immunity and nutrient absorption, are instead significantly reduced in PCOS patients [35,36,37]. The modifications of the gut microbiota in PCOS are different, sometimes controversial and not yet fully understood. However, several studies have tried to investigate the relationship between intestinal microbiota and PCOS; most of these have focused on the connection of intestinal bacteria with insulin-resistance and sex hormones. Table 1 summarizes the effect of changes in major microorganisms in PCOS patients.

### 3.2. Gut Microbiome and Insulin-Resistance

Insulin resistance (IR), together with obesity, is closely related to the gut microbiome as showed in studies conducted on animals and humans [38,39]. After the transplantation of healthy intestinal flora in germ-free mice, an increase in body fat and the development of IR has been observed [40]. Therefore, it can be assumed that, on the contrary, the transplantation of a healthy microbiome in a subject affected by metabolic syndrome determines an improvement in clinical parameters, as was demonstrated by Vrieze et al.: after six weeks from transplantation of gut microbiome of healthy people into patients with metabolic syndrome, an increase in insulin sensitivity and a decrease of body mass index (BMI) of the recipients was observed [40]. The gut microbiome could therefore play a role in the development of IR; the mechanisms that connect IR, gut microbiome and PCOS are manifold and were recently summarized in the review by He et al. [41]. Imbalance of gut microbiota, increasing intestinal permeability, could determine a chronic low-grade inflammation by activating the immune system. Proinflammatory cytokines interfere with insulin receptor function, causing IR/hyperinsulinemia; indeed, it has been founded that with the increased intestinal permeability and the consequent introduction of LPS into the blood circulation of mice and humans, fasting blood glucose and insulin levels increase [13,42,43]. Another possible link between the gut microbiome and IR sees involved gastrointestinal hormones like Ghrelin and peptide YY (PYY), both showing a negative correlation with IR and BMI [25,44,45]. Liu et al. reported a lower level of ghrelin and PYY in women with PCOS compared with those of healthy women, maybe due to an increase in Bacteroides species that are negatively correlated with ghrelin [25]. However, not all studies found differences in ghrelin and PYY levels in women with PCOS compared to healthy women, although gut microbiota may cause altered secretion of these hormones leading to insulin resistance and hyperinsulinemia [46,47]. The relationship between gut microbiota, brain-intestinal axis mediators, and PCOS phenotypes is not yet and sufficiently investigated. To date, it is widely recognized that there is a link between IR in PCOS patients and intestinal dysbiosis. Indeed, as aforementioned, the composition of the gut microbiome in patients with PCOS is significantly altered compared with that of women without PCOS. Zeng et al. were the only ones that investigated the profile of gut microbiome in PCOS patients with IR, compared with that of PCOS patients without IR [35]. They showed differences between the gut microbiome of PCOS patients with IR and without IR. Changes in the intestinal microbiota are much more marked in the forms of PCOS with IR that have the highest level of *Bacteroidaceae* and a greater decrease of Prevotellaceae compared with PCOS women without IR. Moreover, patients with IR displayed a significant difference in the abundance of *Ruminococcaceae* and *Lachnospiraceae* when compared to insulin-sensitive patients. Table 2 summarizes the relationship between changes in major microorganisms and the onset of IR.

### 3.3. Gut Microbiome and Sexual Hormones 

There is ample evidence that sex influences the composition of the gut microbiome. Indeed, the microbiome of women, compared to that of men, is characterized by greater α diversity and a relatively smaller representation of *Bacteroides* [48,49]. Numerous other differences in the microbiota of the two sexes were highlighted. *Prevotella* has a strong positive correlation with testosterone and negative associations with estradiol concentrations, so were more abundant in men than women [50]. Moreover, men had a lower abundance of *Clostridia*, *Methanobrevibacter*, and *Desulfovibrio* compared with women [48,49,50,51]. These differences could be caused by sex chromosomes or by sex hormones; while the first one has not been described, the latter has been extensively evaluated in rat studies that verified the effect of castration on the gut microbiome [52,53]. Sex hormones can determine alterations of the intestinal microbiome by activating the receptors present in the gastrointestinal tract, by altering beta-glucuronidase activity, or by modulating systemic or intestinal immunity [29]. Therefore, it was postulated a relationship, perhaps causal, between the hyperandrogenism of PCOS and the alterations of the intestinal microbiome. The study of Torres et al. using the 16s ribosomal RNA sequencing, analyzed and compared the gut microbiome of 48 healthy women, 42 women with PCOM and 73 women with PCOS [26]. The results has highlighted a reduction in the number of bacterial species (α diversity) and phylogenetic diversity in women with PCOS. Moreover, women with PCOS have also changes in the composition of the microbial community (beta diversity). This evidence was in agreement with those of previous studies [24,25]. Besides, Torres et al. have reached two important pieces of evidence. First of all, they showed that also in women with PCOM occurs alteration in the gut microbiome, which was intermediate between that of healthy control and women with PCOS. Secondly, using multiple and single linear regression analyses, the study showed the inverse correlation between the biodiversity of the microbiome and the hyperandrogenism, rated as serum total testosterone level and hirsutism. Therefore, as testosterone levels increase, there is a reduction in α diversity (*p* = 0.006) and a modification in β diversity (*p* = 0.0009). The study of Insenser el al. has shown a positive correlation between α diversity and testosterone levels and a negative correlation with estradiol concentrations, suggesting that sex hormones might be involved in the modification of gut microbiome, without, however, being able to confirm the causal relationship and the direction of the same (is it the sex hormones that influence the microbiome or, conversely, is it the gut microbiome that influences the hormone levels?) [27]. Moreover, in this study, a difference between men and women also at the genera level was showed [27]. *Raoultella* genus is prevalent in women than men, while *Megasphaera*, *Paraprevotella*, and *Butyricimonas* genera are less frequent. Instead, women with PCOS have an increased abundance of the *Catenibacterium* and *Kandleria* genera compared with men and control women. In conclusion, it might be possible that hyperandrogenemia, characterizing some forms of PCOS, modifies the normal microbiome’s structure leading to an alteration of intestinal permeability which triggers the aforementioned mechanism responsible for IR. Farther, the secretion of androgens and the consequent hyperandrogenemia promoted by IR is able to stimulate decomposition of visceral adipose tissue, leading to an increase in free fatty acids, which further aggravate the levels of IR, creating a vicious cycle that promoting the occurrence and development of PCOS [13,54]. Nevertheless, a clear correlation between hyperandrogenism and the gut microbiome in determining the genesis of PCOS is lacking.

### 3.4. Pathway Leading to PCOS

Though the relationship between the alteration of gut microbiome and PCOS has been found, only few studies have explored the possible mechanisms through which gut microbes are associated with PCOS. Wang et al. (2021) described how the intestinal microbes influence the progression of PCOS by upregulating or downregulating hormone secretion, gut-brain mediators, cytokines, and metabolite production [54]. One of the first examples is related to the role of bile acids, interleukin-22 and *Bacteroides vulgatus* (*B. vulgatus*). As highlighted in the study conducted by Qi et al., it has been reported a significant increase in *B. vulgatus* in PCOS patients compared with healthy controls [31]. Healthy recipient mice transplanted with faecal microbiota from PCOS women or fed *B. vulgatus* develop insulin resistance, a disrupted oestrous cycle and hormone abnormalities, all typical features of PCOS disease: based on this observation, Qi et al. described in the species *B. vulgatus* of individuals with PCOS a significant increase in the abundance of bile salt hydrolase (*bsh*) genes, which encode bile salt hydrolases. As a consequence, in PCOS groups, the levels of glycodeoxycholic acid (GDCA) and tauroursodeoxycholic acid (TUDCA) decreased due to the action of the encoded protein, bile salt hydrolases: thus, an increased abundance of *B.vulgatus* reduced the concentrations of the bile acids, which negatively influenced the production of interleukin-Normally, IL-22 administration is very effective in ameliorating insulin resistance, regulating disrupted oestrous cycles, reversing ovary morphological changes and improving infertility, all typical aspect of PCOS phenotypes. Its role may be explain through two mechanisms: first, an upregulated expression of brown fat-related genes and brown adipose tissue, and second, a resolution of inflammation targeting ovarian granulosa cells. Thus, the decrease of IL-22 was not able to well reverse the PCOS phenotypes, leading to insulin resistance and ovary dysfunction [31]. A further element that can help researchers understand the role of gut microbiome is the evidence of lower concentrations of short-chain fatty acids (SCFA) in the faecal samples of PCOS patients [55]. Indeed, the growth of *Faecalibacterium prausnitzii*, *Bifidobacterium* and *Akkermansia* is promoted by probiotics supplementation, which are SCFA-producing bacteria, and lead to an increase in intestinal SCFAs. These SCFAs bind to their receptors on enteroendocrine cell membranes and directly stimulate the release of gut-brain mediators such as ghrelin and PYY, whose increase is able to influence sex hormone secretion by the hypophysis and hypothalamus through the gut-brain axis, thus exerting an impact on PCOS symptoms [56]. The increased production of SCFAs also contributes to the barrier function of the gut and reduces the translocation of endotoxins across the gut wall, resulting in reduced inflammation and insulin resistance. Finally, there is a potential interaction between sex hormones and gut microbiota, and their interplay may contribute to the pathogenesis of PCOS [36]. The abundance of *Prevotella* is positively related to androgen levels, especially testosterone and androstenedione [57], whereas the abundance of *Kandleria* with the circulating androstenedione concentrations [27]. *Lactobacilli* are also positively correlated with oestradiol and estrone levels as, transplantation of *Lactobacillus* or faecal microbiota from normal rats improve the PCOS phenotypes reducing androgen biosynthesis [36].

## 4. Lower Genital Tract Microbiome in PCOS Patients

Menopause, sex hormones (especially estrogen), age and hygienic habits could influence the composition of lower genital tract (LGT) microbiome since prepuberty and postmenopausal women present different kinds of microbes [58]. The main factors leading to the alteration of LGT microbiome PCOS patients are to be found in the irregular menstruation and abnormal hormone levels [59]. We know that during the normal menstrual cycle estrogen and progesterone causes periodic changes in the epidermal cells of the reproductive tract, which may play a critical role in maintaining the microenvironment of the LGT and considered the menstrual cycle irregularity in PCOS patient, this can strongly alter the composition of PCOS patients’ LGT microbiomes. The vagino-uterine microbiome undergoes small changes between the two phases of the menstrual cycle (proliferative and secretory phase): proliferative period seems to be related to the increase of bacterial proliferation in the vagina and endometrium, which may be the reason why the microorganism in the reproductive tract of PCOS patients is easier to alter [60,61,62]. Moreover, also several studies on rats’ models have demonstrated that rats with PCOS do not show the normal cyclical fluctuations of the vaginal microbiota observed in normal rats [54]. The composition of PCOS patients’ LGT microbiome has not yet been characterized: in 2020, Tu et al. evaluated the LGT microbiome composition of PCOS women, sampling microbiota biopsy of both the vagina and cervical canal of 97 reproductive-aged women [63]. A significant difference of taxa abundance between PCOS and healthy women in both vaginal and cervical canal microbiomes was demonstrated by 16S rRNA gene sequencing on a total of 194 microbial samples. In detail, a great heterogeneity of microbiome composition was observed both in the vagina and cervical canal with a significantly reduced proportion of *Lactobacillus* and, conversely, an increase of several potential pathogenic taxa, such as *Gardnerella vaginalis*, *Chlamydia trachomatis* and *Prevotella*, in PCOS patients. More than one author has found a tight association between the onset of vaginosis, infertility, abortion, stillbirth, preterm labor, recurrent implantation failure, and many other adverse pregnancy outcomes, and reduced levels of *Lactobacillus* spp. in the female reproductive tract [64,65,66,67]. *Gardnerella* and *Prevotella species* have been reported to be closely related to bacterial vaginosis (BV), which is often overlooked and can be difficult to treat because easy relapses and increase woman’s susceptibility to other types of infections, including HIV [65,66,67,68]. *Gardnerella vaginalis* can also be detected in the endometrium of about half of women with BV, and may have adverse effects on the procedure of embryo implantation and even the growth of the fetus [69]. Considering that PCOS women are often plagued by infertility, abortion, fetal arrest, preterm birth, and several other adverse reproductive outcomes, it is questionable whether the composition of microorganisms in PCOS women’s LGT may lead to these adverse reproductive phenotypes. In a case-control study conducted by Hong X et al. [18], the microbiome structure was studied by 16S rRNA gene sequencing using vaginal swabs of 39 women with newly diagnosed PCOS and 40 healthy controls. A in The vaginal bacterial structures was found to be significantly difference in the PCOS women compared to healthy one. The Simpson index for PCOS group versus (vs) control group showed a median of 0.49 vs. 0.80, *p* = 0.008, a Shannon index a median of 1.07 vs. 0.44, *p* = 0.003, while the Chao1 index a median of 85.12 vs. 66.13, *p* < 0.0 *L. crispatus* was significantly lower in the PCOS group (*p* = 0.001), whereas *Mycoplasma* and *Prevotella* was higher in this group when compared to controls (*p* < 0.001, *p* = 0.002, respectively). The ROC analysis highlighted that the area under the curve (AUC) for the relative abundance of Mycoplasma was 0.958 (95% CI: 0.901–0.999) underling *Mycoplasma genus* as a potential biomarker for PCOS screening [18]. Whether the vaginal microbiome impacts the PCOS occurrence or development is still unknown. Normally, disruption in ‘normal’ vaginal equilibrium is defined as BV, which is characterized by increasing pH, epithelial cell destruction and local inflammation [70]. The potential impact of the vaginal microbiome on the female LGT is worth investigating.

## 5. Diet and Medication: How to Change Gut Microbiome

Since the relationship between microbiome and PCOS pathogenesis is getting more and more defined, a link between diet, microbiota, and PCOS could be postulated, revealing the potential impact on prevention and treatment of this condition. To date, the possible relationship with medication is inconsistent. On the other hand, diet could play a therapeutic role for several chronic diseases, especially those related to metabolic syndrome, through the modulation of the microbiome. In their review, Singh RK et al. described the microbiome and its modification through diet, with the influence on inflammatory bowel disease, obesity, type 2 diabetes, cardiovascular disease, cancer and its response immunotherapy [71]. They showed the different impact of animal and plant proteins on gut microbiota: animal-based proteins have been noted to increase *Bacteroides*, *Alistipes* and *Bilophila*, and to reduce counts of *Bifidobacterium (B.) adolescentis*; those changes increased risk of cardiovascular disease. On the opposite, plant proteins have been reported to increase *Bifidobacterium* and *Lactobacillus*, and to decrease *Bacteroides fragilis* and *Clostridium perfringens*, with the positive health outcome of increasing SCFA’s (Short Chain Fatty Acids) levels and consequent decreasing inflammation. The Authors also reported that a high-fat diet increases counts of *Bacteroides* and correlates with less *Lactobacillus intestinalis* and more *Clostridiales*, *Bacteroides*, and *Enterobacteriales*, associated with inflammation. Regarding carbohydrates, it has been suggested that high levels of glucose, fructose, and sucrose increase *Bifidobacteria* and reduce *Bacteroides.* Non-digestible carbohydrates, such as whole grain and wheat bran, seem linked to an increase in *Bifidobacteria* and *Lactobacilli* [71].

Moreover, it has been demonstrated that those dietary patterns can modify the natural history of pathologic conditions, like type 2 diabetes, independently of antidiabetic drugs, with a significant reduction of glucose and improvement of dyslipidemia and inflammation.

This is also confirmed by the evidence that a diet as poor as that of the “fast-food culture” can cause dysbiosis, a condition linked with numerous chronic diseases, as obesity, cardiovascular diseases, and cancer [72,73].

Given this association, there may be significant therapeutic utility for several pathologies in altering microbial composition through diet. Nevertheless, there is only one study investigating if diet could play any protective or therapeutic role in PCOS. Xue et al. demonstrated that a 21-day treatment with inulin and metformin in mice could cause a decrease of ovarian damage and improvement of PCOS, through the increase of *Lactobacillus* and *Bifidobacterium*, the latter with a proven great anti-inflammation capable [74].

Actually, diet and medication contribution to potential therapy or their protective role on disease’s development and progression is currently poor understood and should be further investigated. 

## 6. Therapeutic Opportunities

With a better understanding of the role of the microbiome in PCOS pathogenesis, great efforts have been made to develop new treatment options for the management of PCOS [75,76,77]. Literature focused on probiotics, prebiotics, and symbiotic, and more innovative fecal microbiota transplantation and IL-22 (Table 3).

### 6.1. Probiotic, Prebiotics and Synbiotics

Probiotics are “live micro-organisms that, when administered in adequate amounts, confer a health benefit on the host” according to the World Health Organization (WHO) [78]. Probiotic microorganisms are naturally found in fermented foods: they perform anti-oxygenic, anti-microbial, anti-inflammatory effects, improving metabolic parameters, modulating intestinal microbiota, and regulating the immune system [79]. To administer probiotics as a therapy implies appropriate supplementation with probiotics to treat diseases.

Although the underlying mechanism remains unclear, probiotics therapy has shown a positive effects on the metabolic profile in women with PCOS [79,80,81,82,83,86,87]. Ahmadi et al. reported that probiotic supplementation (*L. acidophilus*, *L. casei*, and *B. bifidum*) for a period of 12 weeks caused a statistically significant decrease in weight and BMI in PCOS patients compared with the placebo, with beneficial effects on glycaemia, triglycerides (TG) and very-low-density lipoprotein (VLDL) cholesterol [79]. Similar results with the supplementation of *L. casei*, *L. acidophilus*, *L. rhamnosus*, *L. bulgaricus*, *B. breve*, *B. longum*, and *Streptococcus thermophiles* was described in women with PCOS for 8 weeks with a significant decrease in plasma glucose and serum insulin levels [80]. Moreover, Rashad et al. found that probiotic supplementation (*L. delbruekii* and *L. fermentum*) for 12 weeks significantly reduced Homeostatic Model Assessment of Insulin Resistance (HOMA-IR) levels, with an additional improvement of lipid profile [81]. The probiotic therapy with *L. acidophilus*, *L. plantarum*, *L. fermentum*, and *L. gasseri* has also evidenced a possible role in modulation of inflammatory processes when administered for 12 weeks in women with PCOS [86].

In a meta-analysis of seven RCTs, Heshmati et al. found no significant effect of probiotic supplementation on anthropometric indices like weight, BMI, and waist circumferences as well as HOMA-IR and LDL, in PCOS patients compared to placebo; they instead found a significant effect on glycemic control, with lower insulin levels, and on lipid metabolism, by lowering TG serum levels and increasing HDL. These data suggest that probiotic supplementation can be applied as an adjunct therapy for PCOS management. The effects of probiotic therapy on the hormonal profile of women with PCOS are not so widespread [82]. In a recent metanalysis, a significant effect of probiotics on the control of hormonal and inflammatory indicators has been reported by Shamasbi et al., with a significant reduction of Free Androgen Index (FAI) and malondialdehyde (MDA), and an increase Sex Hormone Binding Globulin (SHBG) and nitric oxide (NO) [83].

Another study showed that probiotic supplementation of PCOS women for 12 weeks had beneficial effects on total testosterone, SHBG, and MDA levels but did not affect other metabolic profiles [82]. Notable, the probiotics, and their dosages, used in studies varied widely, and that is why future studies will face the standardization of these aspects. 

Regarding prebiotics, the most known ones are fructooligosaccharides (FOS), inulin, galactooligosaccharides (GOS), and lactulose [78]. They alter the composition of the microbiota, performing positive effects on the health of the host.

Some studies have shown that prebiotics has positive effects on metabolic markers and immunomodulatory properties because they induce the growth of both *Bifidobacterium* and *Lactobacillus*, which in turn produce an important reduction in fasting plasma glucose, serum TG, total cholesterol, and LDL cholesterol, and a significant increase in HDL cholesterol levels [87]. Fernandes et al., in this sense, indicated that some prebiotics directly controlled hyperglycemia and HOMA-IR [88]. These results may indicate that the presence of prebiotics, along with probiotics, improves their effectiveness in reducing LDL. Moreover, probiotics (or synbiotics) may decrease triglyceride level (mean difference (MD) −17.51 mg/dL, 95% CI −29.65 to −5.36) [82]. The androgen levels, hirsutism, and menstrual cycle irregularities could also improve by the resident dextrin consumption in women with PCOS [84].

However, additional researches are needed to elucidate and compare the efficacy of several probiotic strains and various doses of probiotics, to identify the proper duration of treatment, and to demonstrate the health benefits of probiotics, prebiotics and synbiotics on clinical outcomes in PCOS [84].

### 6.2. Fecal Microbiota Transplantation

Fecal microbiota transplantation (FMT) consists of the infusion of microorganisms from the feces of healthy donors to a recipient’s small intestine, in order to rapidly change the composition of the new hostʼs gut microbiome and treat diseases [85]. It could represent a potential innovative treatment option for PCOS. 

In their study, Guo Y et al. reported how microbiota interventions through FMT and *Lactobacillus* transplantation in PCOS-induced rats had a beneficial effect; indeed, this kind of treatment results to be associated with a decrease in serum androgen levels and an increase in estrogen levels, leading to a regulated menstrual cycle [36]. Guo Y et al. showed metabolic improvements in FMT-treated PCOS rats vs. the untreated group, with decreased androgen levels, estradiol and estrone increase and normalization of ovarian function [36].

However, to date, there are no clinical reports on the use of FMT to treat PCOS except murine models; prospective data obtained from laboratory researches should encourage further studies on humans [89].

### 6.3. New Therapeutic Options (IL-22)

The therapeutic role of intestinal immune factor IL-22 has been studied in PCOS-induced mice models, but it is still not so clear. Qi et al. tried to explore the possible mechanism of IL-22 in regulating hyperandrogenism-associated PCOS, and they found that additional IL-22 treatment in PCOS-induced mice improved insulin-resistance, estrous cycle and ovary morphology. They hypothesized that IL-22 upregulated the browning of white adipose tissue, resulting in regulated insulin sensitivity and ovarian functions in women affected by PCOS with a hyperandrogenism phenotype [90]. 

Moreover, Qi et al. reported low levels of IL-22 in individuals with PCOS, mediated by reduced IL-22 secretion induced by glycodeoxycholic acid; indeed, reduced glycodeoxycholic acid and tauroursodeoxycholic acid levels were found after transplantation of fecal microbiota from women with PCOS to recipient mice, resulting in insulin resistance, altered bile acid metabolism, reduced interleukin-22 secretion, worst ovarian functions and infertility [31]. 

These results suggest that modifying the gut microbiota and administrating IL-22 exogenous may represent the effective treatment of PCOS [91].

## 7. Conclusions

Diversity enriches: starting from this simple sentence and moving the concept to PCOS, the dysbiosis of gut microbiota that occurs in PCOS lead to a decrease of diversity and the abundance of some bacteria species causes metabolic disorders.

Current evidence stressed the contribution of the gut microbiome in conditions unrelated to the gut and emerging researches demonstrated its involvement in PCOS, starting from the observation that humanized mice receiving a fecal transplant from women with PCOS develop ovarian dysfunction, along with immune changes and insulin resistance.

To date, the causal contribution of gut microbiota composition and disruption in secondary bile acid biosynthesis in the pathogenesis of PCOS seems to be proven. As discusses in this paper, the proof that the administration of IL-22 and bile acid glycodeoxycholic acid improved insulin resistance, ovarian dysfunction and infertility in mice with PCOS, suggests that gut microbiota may have a causal role in mediating disrupted insulin sensitivity and ovarian function in PCOS. In light of this evidence, it is possible to believe that a future therapeutic approach for PCOS may involve gut microbiota.

The supplementation with prebiotic, probiotic, and synbiotic in women with PCOS seems to improve many biochemical findings and beneficially affect but the mechanism is still unclear. Further studies are needed to establish the role of these agents in PCOS treatment or, maybe, prevention.

Future prospective and, especially, randomized clinical studies are needed to explain mechanisms underlying this association, the causes of dysbiosis of gut microbiota, and the role of the gut microbiota in PCOS.

## Figures and Tables

**Table 1:** The effect of changes in major microorganisms in PCOS patients.

PCOS	
Microorganisms	Effect
Increase of Escherichia and Shigella	Altered production of short-chain fatty acids
Increase of Bacteroides vulgatus	Reduction in the levels of glycodeoxycholic and tauroursodeoxycholic acid
Decrease of Prevotellaceae	Loss of production of anti-inflammatory metabolites
Decrease of Lactobacilli and Bifidobacteria	Reduced immunity and nutrient absorption

**Table 2:** The relationship between changes in major microorganisms and the onset of IR.

Insuline Resistance (IR)	
Microorganisms	Effect
Imbalance of gut microbiota (significant difference in the abundance of Ruminococcaceae and Lachnospiraceae)	Increased intestinal permeability → chronic low-grade inflammation by activating the immune system → production of proinflammatory cytokines interfere with insulin receptor function → IR/hyperinsulinemia
Increase of Bacteroides species	Altered secretion of Ghrelin and peptide YY → IR/hyperinsulinemia.

**Table 3:** Therapeutic opportunities acting on the gut microbiome (HDL: High-Density Lipoprotein; LDL: Low-Density Lipoprotein; TG: Triglyceride; FMT: Fecal Microbiota Transplantation).

Therapy	Studied Model	Effects	Reference
Probiotic	Human	Positive effect on glycemic control, with lower insulin levels, and on lipid metabolism, by increasing HDL and lowering TG serum levels; positive control of hormonal and inflammatory indicators	[76,77,78,79,80,81]
Prebiotics	Human	Positive effects on metabolic markers and immunomodulatory properties; considerable decrease in fasting plasma glucose, serum TG, total cholesterol, and LDL cholesterol, and significant increase in HDL cholesterol levels.	[75,82,83]
FMT	Mice	Metabolic improvements in FMT-treated PCOS rats vs. the untreated group, with decreased androgen levels, estradiol and estrone increase and normalization of ovarian function.	[36]
IL-22	Mice	Improved insulin-resistance, estrous cycle and ovary morphology.	[84,85]

## Data Availability

Data available on request.

## Abbreviations

PCOS	polycystic vary syndrome
HA	hyperandrogenism
NIH	National Institutes of Health
ESHRE/ASRM	European Society for Human Reproduction and Embryology/American Society for Reproductive Medicine
PCOM	polycystic ovarian morphology
DOGMA	dysbiosis of gut microbiota
LPS	lipopolysaccharide
MeSH	medical subject headings
IR	insulin resistance
BMI	body mass index
PYY	peptide YY
GDCA	glycodeoxycholic acid
TUDCA	tauroursodeoxycholic acid
SCFA	short-chain fatty acids
LGT	lower genital tract
BV	bacterial vaginosis
AUC	area under the curve
WHO	World Health Organization
TG	triglycerides
VLDL	very-low-density lipoprotein
HOMA-IR	Homeostatic Model Assessment of Insulin Resistance
FAI	Free Androgen Index
MDA	malondialdehyde
SHBG	sex hormone binding globulin
NO	nitric oxide
FOS	fructooligosaccharides
GOS	galactooligosaccharides
FMT	fecal microbiota transplantation
